# Supplementary material for: The relationship between environmental sources and the susceptibility of Acanthamoeba keratitis in the United Kingdom
Source: PLoS One. 2020 Mar 11;15(3):e0229681. doi: 10.1371/journal.pone.0229681 (PMC7065798; doi:10.1371/journal.pone.0229681)
Supplement: S1 Data — (DOCX) [file pone.0229681.s001.docx]

## **HOUSEHOLD WATER**

**On a morning BEFORE a tap is turned on, open the tube with the white cap labelled for the corresponding tap (bathroom or kitchen).**

**SPOUT (BATHROOM AND KITCHEN)**

| 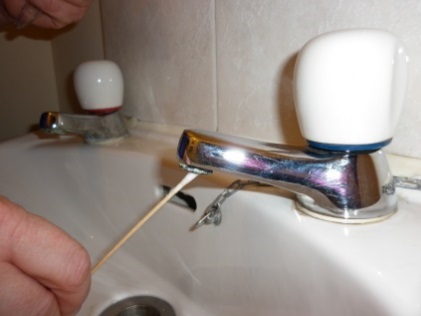 | | **Step 1**  Peel open the swab, holding it at the end, rub for 10 seconds around the inside of the tap **SPOUT**. |
| --- | --- | --- |
| 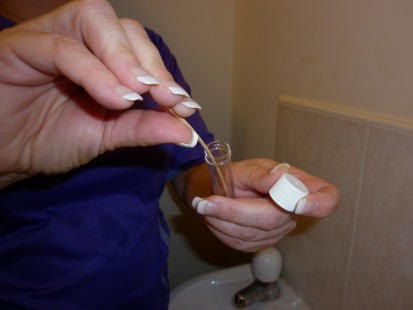 | | **Step 2**  Place the swab in the tube and break off the end. |
| 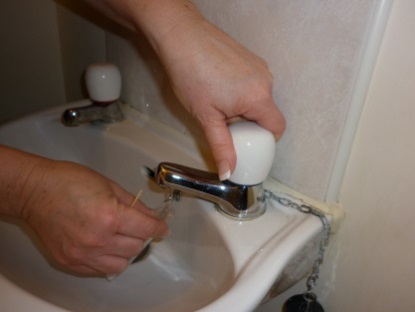 | **Step 3**  Softly turn on the COLD tap and drizzle a small amount of water into the tube containing the swab. | |
| 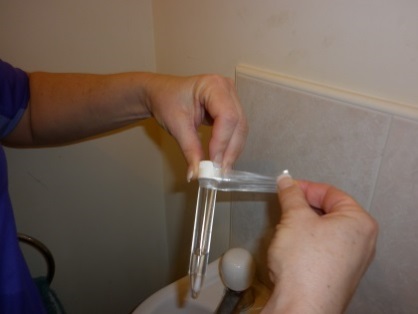 | **Step 4**  Close the lid and stretch the section of parafilm around the tube seal to prevent leakage. | |

**PLEASE TURN OVER**

## **HOUSEHOLD WATER**

***For the drain and overflow, please repeat the same procedure as before***

**DRAIN (BATHROOM AND KITCHEN)**


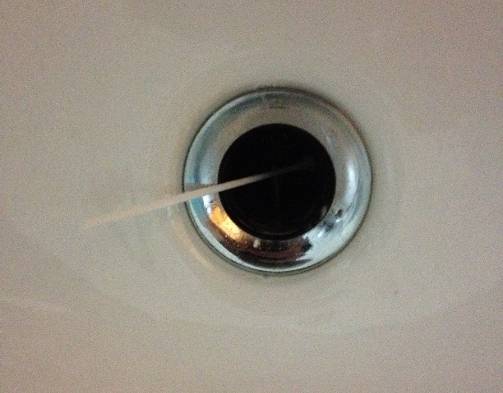


**1.** Peel open the swab, holding it at the end, rub for 10 seconds around the inside of the **DRAIN**.

**2.** Place the swab in the tube and break off the end.


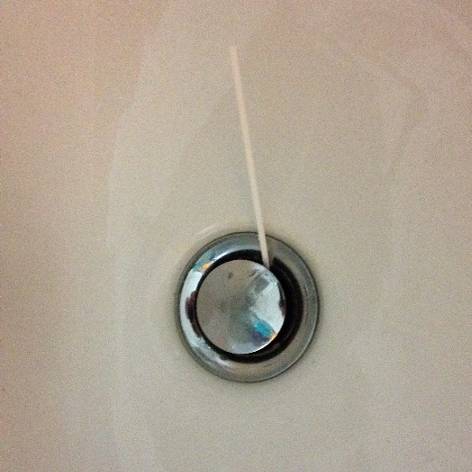
**3.** Softly turn on the COLD tap and drizzle a small amount of water into the tube containing the swab.

**4.** Close the lid and stretch the section of parafilm around the tube seal to prevent leakage.

**OVERFLOW (BATHROOM AND KITCHEN)**

| 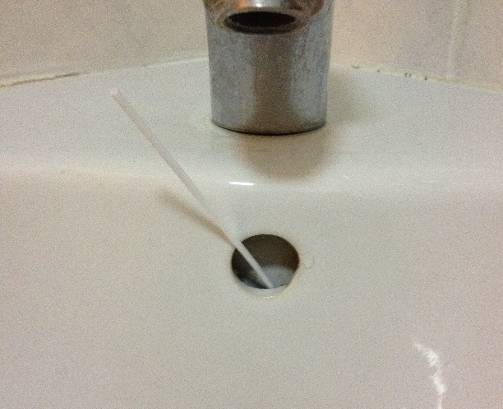 | **1.** Peel open the swab, holding it at the end, rub for 10 seconds around the inside of the **OVERFLOW**.  **2.** Place the swab in the tube and break off the end.  **3.** Softly turn on the COLD tap and drizzle a small amount of water into the tube containing the swab.  **4.** Close the lid and stretch the section of parafilm around the tube seal to prevent leakage. |
| --- | --- |

Place the tubes back in the plastic bag and send in the postage paid envelope as soon as possible after collection.

Dispose of the packaging in your normal garbage waste.

**Thank you for your support of this research.**
