# Supplementary material for: The relationship between environmental sources and the susceptibility of Acanthamoeba keratitis in the United Kingdom
Source: PLoS One. 2020 Mar 11;15(3):e0229681. doi: 10.1371/journal.pone.0229681 (PMC7065798; doi:10.1371/journal.pone.0229681)
Supplement: Attachment — Submitted filename: The relationship between environmental sources and the susceptibility of Acanthamoeba keratitis in the United Kingdom.Carnt.2019.docx [file pone.0229681.s002.docx]

The relationship between environmental sources and the susceptibility of *Acanthamoeba* keratitis in the United Kingdom.

Nicole Carnt – 2019, reviewed Dec 2019.

Major changes

- As your study was designed to search for *Acanthamoeba* and then record the presence of other FLA I would switch this throughout the text. “To determine whether *Acanthamoeba* keratitis (AK) patients have higher rates of *Acanthamoeba* or other free-living amoeba (FLA) colonising domestic sinks than control contact lens (CL) wearers, and whether these isolates are genetically similar to the corneal isolates identified from their CL associated AK.”
- There is no description of the other FLA identified, can you describe which others were investigated and found?
  - Can this also be broken down within the bar graphs?
- The quality of all of the figures need to be improved (use graph pad prism or another software for better images).

Figure 1.

- Do these graphs describe the full picture? Although the graphs tell the reader total numbers it doesn’t break down the exact number of samples obtained from the spout from the kitchen of an AK wearer or the drain from the bathroom of a CL control as examples. A reorganization and coloration to be more descriptive may be useful.

Figure 2.

- As you are specifically identifying different FLA, this graph could be a stacked bar graph with different colors representing the different genus of FLA found for both groups and areas swabbed.

Minor Changes

- Lines 19-21, 37-41, 79, 113 – switch *Acanthamoeba* and FLA order.
- Don’t you think using *E. coli* and 32°C selects for *Acanthamoeba* over other FLA?
- There was no discussion to where the patients were from within the UK (distribution, were these all local to a particular area? – This data should be easily accessible since collected during the questionnaire). Can you make a conclusion about geographic location, potential differences in water storage methods, sources that the water was from or treatment methods? You may find that there was a particular area that could have an increased risk or contamination. This could be useful for water regulatory and treatment of such bodies of water.
- Why were only 4 patients out of 129 compared for Ac *coxA*? Please discuss.
- Lines 24-26 – “The samples were cultured by inoculating onto a non-nutrient agar plate seeded with *Escherichia coli* and the plates were incubated at 32˚C and examined for amoebae by microscopy for up to 2 weeks.” Could read as “The samples were cultured by inoculating onto a non-nutrient agar plate seeded with *Escherichia coli*, incubated at 32˚C and examined for amoebae by microscopy for up to 2 weeks.”
- Lines 30-36 – Please add the %’s for spouts, drains, overflow in kitchens and bathrooms.
- Line 36 – Was this the kitchen or bathroom sink?
- Line 46 – Change “potentially” to opportunistically. Where these the different types of FLA identified within this study? Please make reference to which ones?
- Line 71 – Reference to ref 11 not needed here since it is referenced at the end of the sentence.
- Line 95-96 – If 23x repeat samples were taken from 129 patients from 6 locations within the home there would be 17,802 samples? I would remove this sentence. Or please show the succession of each patient over the 23 month period as a supplemental figure.
- Line 114 – 513 total samples were obtained from AK patients = 6.6 samples per patient? 189 samples from 40 CL controls = 4.7 samples per patient?

Figure 1.

- Please add in total numbers for each of the described %’s within the pie chart.
- Please add discussion about why the 1.17% (6 cases) from the Cloakroom of AK patients was removed.
- Line 121 – (The *Acanthamoeba* comparison should be Figure 2 not “Fig 1”. Also spell out figure to keep consistent throughout the MS.
- Line 123-124 – Do both groups (AK patients and CL control) FLA total include the *Acanthamoeba* data as well? If so, shouldn’t this be removed/normalized?

Figure 2.

- Please shorten the significance bar for the ** between the FLA found in patients and controls.
- Please keep consistency between titles of groups for all figures, “AK patients” and “CL controls”.
- Line 131-137 – Please include the * for level of significance.
- Line 133-137 – Please spell out figure to keep consistency throughout the MS.
- Line 142 – Please Italicize *Acanthamoeba*.

Figure 3&4.

- Please keep consistency between titles of groups for all figures, “AK patients” and “CL controls”.
- Please shorten the significance bar and center the * for kitchen drain and bathroom overflow.

Table 1.

- Please centers align and middle aligns text into the center of all boxes of the table.
- References font changes.

Comments to reviewers

Line 138-139 – Spouts have the fastest flowing and largest volume of water flowing through it with thousands of gallons per month, this may be the reason why you do not see as much bacteria/*Acanthamoeba*/FLA in that particular area for Fig 3. Should repeat samples be taken from the drain or overflows from these areas for future studies? Please comment/discuss this finding.

Do you think that paranoia of patients when sampling they didn’t want to see *Acanthamoeba* or FLA within their home that they may have stringently cleaned between sampling?

All figures/graphs are of poor quality please use another software program.

Were attempts to characterize the species/isolate causing the AK in these 4 patients?

Supplemental information is clear for samplers.

Molecular characterization of FLA should become mandatory in future studies.
